# Identification of an amino-terminus determinant critical for ryanodine receptor/Ca^2+^ release channel function

**DOI:** 10.1093/cvr/cvaa043

**Published:** 2020-02-20

**Authors:** Monika Seidel, Camille Rabesahala de Meritens, Louisa Johnson, Dimitris Parthimos, Mark Bannister, Nia Lowri Thomas, Esizaze Ozekhome-Mike, Francis Anthony Lai, Spyros Zissimopoulos

**Affiliations:** 1 Department of Cardiology, School of Medicine, Wales Heart Research Institute, Cardiff University, Heath Park, Cardiff CF14 4XN, UK; 2 Swansea University Medical School, Institute of Life Science, Swansea SA2 8PP, UK; 3 Division of Cancer & Genetics, School of Medicine, Cardiff University, Cardiff CF14 4XN, UK; 4 School of Pharmacy & Pharmaceutical Sciences, Cardiff University, Cardiff CF10 3NB, UK; 5 College of Medicine, QU Health, and Biomedical Research Centre, Qatar University, Doha, Qatar

**Keywords:** Arrhythmia, Calcium signalling, Disease mechanism, Excitation-contraction coupling, Ryanodine receptor

## Abstract

**Aims:**

The cardiac ryanodine receptor (RyR2), which mediates intracellular Ca^2+^ release to trigger cardiomyocyte contraction, participates in development of acquired and inherited arrhythmogenic cardiac disease. This study was undertaken to characterize the network of inter- and intra-subunit interactions regulating the activity of the RyR2 homotetramer.

**Methods and results:**

We use mutational investigations combined with biochemical assays to identify the peptide sequence bridging the β8 with β9 strand as the primary determinant mediating RyR2 N-terminus self-association. The negatively charged side chains of two aspartate residues (D179 and D180) within the β8–β9 loop are crucial for the N-terminal inter-subunit interaction. We also show that the RyR2 N-terminus domain interacts with the C-terminal channel pore region in a Ca^2+^-independent manner. The β8–β9 loop is required for efficient RyR2 subunit oligomerization but it is dispensable for N-terminus interaction with C-terminus. Deletion of the β8–β9 sequence produces unstable tetrameric channels with subdued intracellular Ca^2+^ mobilization implicating a role for this domain in channel opening. The arrhythmia-linked R176Q mutation within the β8–β9 loop decreases N-terminus tetramerization but does not affect RyR2 subunit tetramerization or the N-terminus interaction with C-terminus. RyR2^R176Q^ is a characteristic hypersensitive channel displaying enhanced intracellular Ca^2+^ mobilization suggesting an additional role for the β8–β9 domain in channel closing.

**Conclusion:**

These results suggest that efficient N-terminus inter-subunit communication mediated by the β8–β9 loop may constitute a primary regulatory mechanism for both RyR2 channel activation and suppression.

## Graphical Abstract

**Figure:**
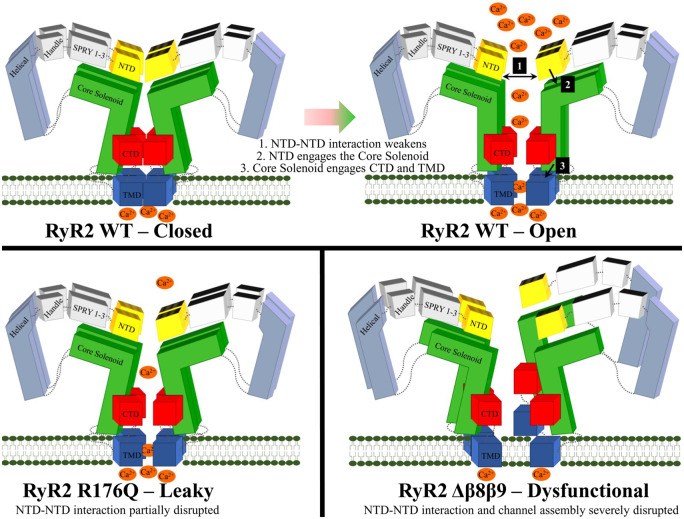

## 1. Introduction

The ryanodine receptor (RyR), a homotetramer whose subunits each consists of ∼5000 amino acids, mediates sarcoplasmic reticulum (SR) Ca^2+^ release to enable excitation–contraction coupling.^1^ In mammalian adult heart, RyR2 is activated by Ca^2+^ influx through L-type voltage-gated channels acting as a Ca^2+^ signal amplifier to initiate sarcomere contraction.^2^ Abnormal RyR2 gating perturbs cardiomyocyte Ca^2+^ handling resulting in acquired and inherited arrhythmogenic cardiac disease.^3^^,^^4^ The recent determination of the RyR1 and RyR2 structures at near-atomic resolution by electron cryomicroscopy has been a major scientific breakthrough in the study of the Ca^2+^ release channel.^5–10^ For the first time, it has been possible to visualize not only secondary structural elements of the peptide backbone but also the position of amino acid side chains for the majority (∼70%) of the RyR1/2 molecular mass. Structural comparison of the channel’s open and closed states reveals that RyR regulation is governed by extensive inter- and intra-subunit interactions. Among them, N-terminal inter-subunit interactions appear to be of paramount importance for the gating of the channel, a phenomenon also indicated by X-ray crystallography/computational docking^11^^,^^12^ and demonstrated by biochemical/functional observations.^13–15^

Here, we describe inter-domain contact sites and assess their role in the regulation of the RyR2 channel. We propose that N-terminus self-association is the gatekeeper of RyR2 channel activity, instrumental in both the pore’s opening and closing mechanisms. Robust N-terminal inter-subunit interactions maintain the wild-type channel closed, whereas modest disruption of N-terminus tetramerization results in a hypersensitive channel as seen with the pathogenic R176Q mutation. Severe disruption of N-terminus tetramerization due to deletion of the β8–β9 loop, which also leads to impaired RyR2 tetramers, results in hyposensitive channels.

## 2. Methods

### 2.1 Materials

The human embryonic kidney (HEK) 293 cell line was obtained from ATCC^®^ (CRL-1573), mammalian cell culture reagents and Fluo-3 AM were obtained from Thermo Scientific, CHAPS from Merck, protease inhibitor cocktail (Complete^TM^) from Roche, nProtein-A Sepharose from GE Healthcare, electrophoresis equipment and reagents from Bio-Rad, enhanced chemiluminescence detection kit from Thermo Scientific, DNA restriction endonucleases from New England Biolabs, Pfu DNA polymerase from Promega, side-directed mutagenesis kit (QuikChange II XL) from Agilent Technologies, T7 Gene 6 Exonuclease from Affymetrix, oligonucleotides, and all other reagents from Sigma unless otherwise stated.

### 2.2 Plasmid construction

The plasmid encoding for wild-type RyR2 N-terminus (NT, residues 1–906) tagged with the cMyc epitope at the N-terminus has been described previously.^15^ The four-alanine substitution within the β13–β14 loop and the small three-residue deletion within the β23–β24 loop (NT^β13^^–^^β14/4Ala^ and NT^Δβ23^^–^^β^24, respectively) were generated in NT using the side-directed mutagenesis QuikChange II XL kit and the complementary primers listed in Supplementary material online, *Table S1*. Larger deletions (within the β8–β9, β20–β21 and β30–β31 loops) were generated using PCR amplification as described elsewhere.^16^ Briefly, primers were designed to contain four consecutive phosphorothioate residues located 12 nucleotides from the 5’end, which enabled digestion with T7 Gene 6 Exonuclease, thereby converting the blunt ends of the PCR product into 12-nucleotide 3’-end overhangs. In addition, the six outermost nucleotides of each primer were complementary to the six nucleotides of the reverse primer located immediately before the phosphorothioate residues, which resulted in self-circularization of the PCR product following exonuclease digestion. PCR was carried out using the side-directed mutagenesis kit, and the PCR product, firstly digested with DpnI followed by digestion with T7 Gene 6 Exonuclease, was transformed into bacteria to obtain plasmid DNA. Plasmid encoding for human RyR2^Δβ8^^–^^β^9 was prepared by replacing a SpeI–BstEII ∼3.8 kb fragment into the WT plasmid, whereas RyR2^R176Q^ plasmid has previously been described.^17^ An RyR2 C-terminal fragment (residues 3529–4967) was generated by PCR amplification from full-length human RyR2 cDNA and cloned into pCR3 (Thermo Scientific, Loughborough, UK) containing an N-terminal HA epitope tag (HA-RyR2-CT). All plasmid constructs were verified by direct DNA sequencing.

### 2.3 Chemical cross-linking

HEK293 cells were transiently transfected with plasmid DNA encoding for RyR2 NT constructs using TurboFect (Thermo Scientific, Loughborough, UK) according to the provider’s instructions. In 24 h post-transfection, cells were homogenized on ice in homogenization buffer [5 mM HEPES, 0.3 M sucrose, 10 mM dithiothreitol (DTT), pH 7.4] by 20 passages through a needle (0.6 mm × 30 mm) and dispersing the cell suspension through half volume of glass beads (425–600 microns, Sigma). Cell nuclei and glass beads were removed by centrifugation at 1500 × *g* for 5 min at 4°C. The supernatant obtained following a subsequent centrifugation step (20000 × *g* for 10 min at 4°C) was retained and protein concentration was measured using the bicinchoninic acid (BCA) colorimetric assay (Thermo Scientific, Loughborough, UK). Cell homogenate (20 μg) was incubated with glutaraldehyde (0.0025% or 260 μmol/L) for the following time-points: 0 min, 2 min, 5 min, 10 min, 15 min, 20 min, 30 min, and 1 h. The reaction was stopped with the addition of hydrazine (2%) and SDS-PAGE loading buffer (60 mM Tris, 2% SDS, 10% glycerol, 5 mM EDTA, 0.01% bromophenol blue, pH 6.8). Samples were analysed by SDS-PAGE and immunoblotting with Ab^cMyc^ (mouse 9E10, Santa Cruz Biotechnology; used at 1:1000 dilution). Tetramer to monomer ratio was determined by densitometry using a GS-900 Scanner (Bio-Rad, Watford, Hertfordshire, UK) and Image Lab software (Bio-Rad, Watford, Hertfordshire, UK). Tetramer formation was calculated as follows: T = OD_T_/(OD_T_ + OD_M_) × 100, where OD_T_ and OD_M_ correspond to optical density obtained for tetramer and monomer bands respectively. Statistical analysis was carried out with GraphPad Prism software using Kruskal–Wallis test with Dunn’s multiple comparison test.

### 2.4 Co-immunoprecipitation

HEK293 cells were transiently co-transfected with plasmid DNA for HA-RyR2-CT (residues 3529–4967 tagged with HA epitope) together with RyR2 NT constructs (residues 1–906 tagged with cMyc epitope) using TurboFect. In 24 h post-transfection cells were homogenized on ice in buffer (20 mM Tris, 150 mM NaCl, pH 7.4) as described above. Cell nuclei and glass beads were removed by centrifugation at 1500 × *g* for 5 min at 4°C and the supernatant was incubated overnight at 4°C in the presence of 0.5% CHAPS under rotary agitation. Following overnight solubilization and centrifugation at 20 000 × *g* for 10 min at 4°C to remove the insoluble material, the supernatant was incubated at 4°C for 6 h with protein A sepharose beads (GE Healthcare) and 2 μg of Ab^HA^ (rabbit ab9110, Abcam) under rotary agitation [2 μg of normal, non-immune rabbit IgG (Santa Cruz Biotechnology) was used as negative control]. Beads were recovered at 1500 × *g* for 2 min at 4°C, washed two times (10 min at 4°C) with the IP buffer (20 mM Tris, 150 mM NaCl, 0.5% CHAPS, pH 7.4) and proteins were eluted with SDS-PAGE loading buffer. A small amount (1/10th) of the IP samples was analysed by SDS-PAGE and immunoblotting with Ab^HA^ (mouse 16B12, Biolegend; used at 1:1000 dilution) to assess HA-RyR2-CT expression and immunoprecipitation. The rest (9/10th) of the IP samples was analysed by SDS-PAGE and immunoblotting with Ab^cMyc^ (mouse 9E10, Santa Cruz Biotechnology; used at 1:1000 dilution) to assess the amount of the co-precipitated RyR2 NT construct. The amount of co-precipitated RyR2 NT proteins was determined by densitometry (using GS-900 Scanner and Image Lab software), normalized against the amount of the input protein in the lysate and specific binding was calculated by subtracting the non-immune IgG IP signal from the anti-HA IP signal. Statistical analysis was carried out using one-way analysis of variance with Bonferroni’s multiple comparisons test.

### 2.5 Sucrose density gradient centrifugation

HEK293 cells transiently transfected with full-length human RyR2 plasmid DNA using TurboFect were harvested after 24 h. Cells were resuspended (10^6^ cells/mL) in hypo-osmotic homogenization buffer (20 mM Tris, 1 mM EDTA, pH 7.4, supplemented with protease inhibitors) and homogenized on ice by 20 passages through a needle (0.6 mm × 30 mm). Cell nuclei and unbroken cells were removed by centrifugation at 1500 × *g* for 5 min at 4°C and the supernatant was subjected to centrifugation at 100 000 × *g* for 1 h at 4°C in order to obtain the microsomal fraction. Protein content was measured using the BCA assay and microsomes were solubilized for 1 h at 4°C in high-salt buffer (1 M NaCl, 0.15 mM CaCl_2,_ 0.1 mM EGTA, 25 mM PIPES, 0.6% CHAPS, 0.3% phosphatidylcholine, 2 mM DTT, pH 7.4, supplemented with protease inhibitors) at a protein concentration of 2.5 mg/mL. The insoluble material was removed by centrifugation at 16 000 × *g* for 10 min at 4°C and the supernatant was layered onto a continuous (5–40%) sucrose density gradient prepared in buffer (300 mM NaCl, 25 mM Tris, 50 mM HEPES, 0.3 mM EGTA, 0.1 mM CaCl_2_, 0.3% CHAPS, 0.15% phosphatidylcholine, 2 mM DTT, pH 7.4, supplemented with protease inhibitors). The gradient was spun at 100 000 × *g* for 16 h at 4°C, fractions were collected, and sucrose concentration was measured using a refractometer. Protein distribution was analysed by SDS-PAGE and immunoblotting with RyR2 Ab^1093^ (raised against human RyR2 residues 4454–4474; used at 1:500 dilution) characterized previously.^17–19^ Densitometry analysis was performed (using GS-900 Scanner and Image Lab software) and the amount of RyR2 in each fraction was normalized against the amount of input protein in the microsomes.

### 2.6 [^3^H]ryanodine binding

[^3^H]ryanodine binding was performed on HEK293 microsomes expressing RyR2^WT^ or RyR2^Δβ8^^–^^β^9 in the presence of 100 μM CaCl_2_ and 10 mM caffeine to promote maximum channel activation. 200 μg of HEK293 microsomes were incubated at 37°C for 2 h in the presence of 8 nM [^3^H]ryanodine (Perkin-Elmer, Seer Green, Buckinghamshire, UK) in binding buffer (25 mM PIPES, 1 M KCl, 2 mM DTT, pH 7.4). Samples were vacuum filtered through glass-fiber filters (GF/F Whatman), incubated overnight in scintillation liquid (Ultima Gold, Perkin Elmer), and radioactivity (dpm) was quantified using a scintillation counter (Tri-Carb 2100 TR, Packard BioScience). Specific binding was calculated from total by subtracting non-specific binding (in the presence of 10 μM non-radiolabelled ryanodine) from three separate experiments each performed in duplicate.

### 2.7 Calcium imaging

Single-cell Ca^2+^ imaging of HEK293 cells expressing RyR2 was adapted from an assay developed by Chen *et al*.^20^ Cells (∼1 × 10^5^) were seeded on poly-lysine coated glass bottom dishes (MatTek, Bratislava, Slovakia) and transiently transfected with plasmid DNA for full-length human RyR2 using Effectene (Qiagen, Manchester, UK) according to the manufacturer’s instructions. After 48 h, cells were loaded with Fluo-3 AM (10 μM) for 1 h at 30°C and immersed in buffer (120 mM NaCl, 25 mM HEPES, 5.5 mM glucose, 4.8 mM KCl, 1.3 mM CaCl_2_, 1.2 mM KH_2_PO_4_, 1.2 mM MgCl_2_, pH 7.4) for imaging at 37°C. RyR2-mediated spontaneous Ca^2+^ release events were monitored using a laser scanning confocal microscope (Leica SP5) and LAS-AF software (Leica Microsystems, Milton Keynes, UK) with the following parameters: ×20 magnification objective lens, excitation at 488 nm and fluorescence emission detected at 500–550 nm, 512 × 512 pixel resolution, 100 ms time interval and scanning speed of 400 Hz. Cells were imaged for 3 min (5 min when thapsigargin was applied) and acquired regions of interest representing global Ca^2+^ environments (typically ∼50 μm^2^) were selected. A broad range of parametric values was calculated from experimental traces by home developed MATLAB (MathWorks) based software. Parameters include oscillatory amplitude, transient duration, frequency of events, caffeine response (10 mM caffeine-induced Ca^2+^ transient typically taken as indication of Ca^2+^ store content), and response to (1 μM) thapsigargin. The store Ca^2+^ content was calculated as the integral of the experimental time-series following administration of caffeine and thapsigargin. To account for the prolonged tail at the end of the measurement, an exponential decay function was fitted on each trace in order to evaluate the total Ca^2+^ release from stores. Statistical analysis was performed using Mann–Whitney test.

## 3. Results

### 3.1 Identification of the N-terminus inter-subunit contact sites

**Figure 1:**
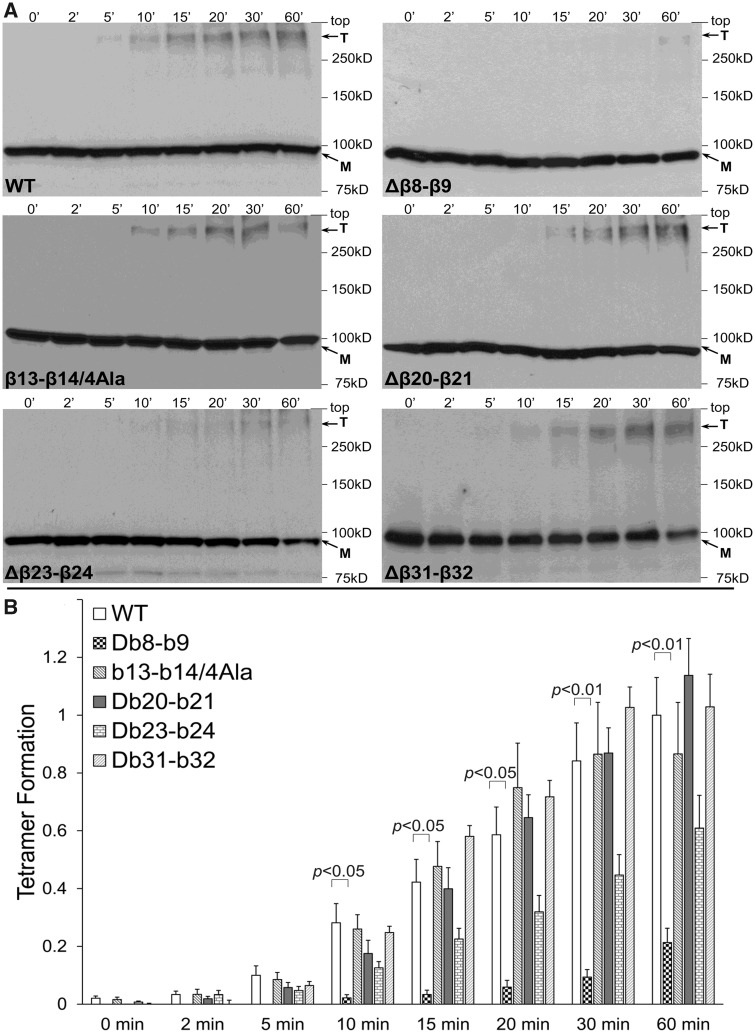
Deletion of the β8–β9 loop perturbs RyR2 N-terminus tetramerization. Chemical cross-linking assays of HEK293 cell homogenates expressing NT (RyR2 residues 1–906) WT or deletion mutants: NT^Δβ8–β9^ (residues 167–178 removed), NT^β13–β14/4Ala^ (residues 240–243 substituted by alanine), NT^Δβ20–β21^ (residues 335–358 removed), NT^Δβ23–β24^ (residues 399–401 removed), and NT^Δβ31–β32^ (residues 748–752 removed). (*A*) Cell homogenates were incubated with glutaraldehyde for the indicated time points under reducing (10 mM DTT) conditions and analysed by western blotting using Ab^cMyc^; monomer (M) and tetramer (T) are indicated with the arrows. (*B*) Densitometry analysis (*n* ≥ 6) was carried out on the bands corresponding to tetramer and monomer moieties and used to calculate tetramer formation. Data are given as mean value ± SEM; statistical analysis was carried out using Kruskal–Wallis test with Dunn’s multiple comparison test. SEM, standard error of the mean.

We have previously found that the RyR2 N-terminus (NT, residues 1–906) displays robust self-association shown by yeast two-hybrid, co-immunoprecipitation (co-IP), gel filtration, and chemical cross-linking assays.^13–15^ Previous X-ray crystallography and computational docking studies placed the four N-terminal domains immediately adjacent to each other at the centre of full-length RyR1^11^^,^^12^ enabling visualization of putative contact sites. In particular, the peptide sequence connecting the β8 with the β9 strand (β8–β9 loop) on one subunit is in close physical proximity with the β23–β24 loop and possibly with the β13–β14 loop (which was not resolved in the crystal structure) on the adjacent subunit. Furthermore, the β20–β21 loop on one subunit may be within contact distance with the β20–β21 loop of a neighbouring subunit. In order to empirically test the involvement of these sequences in RyR2 N-terminus tetramerization, we generated a number of discrete NT constructs with targeted small internal deletions for use in cross-linking assays (Supplementary material online, *Table S2*). To ensure that the overall folding of the NT peptide is unaffected, deletion constructs did not involve complete removal of all the amino acids within the targeted loop but retained some residues sufficient to bridge the adjoining β sheets. In the case of the short four-residue β13–β14 loop, removal of any of these four amino acids could potentially alter the local conformation of the surrounding β sheets and, therefore, a four-alanine substitution rather than deletion was generated. NT deletion constructs were expressed in HEK293 cells and reacted with glutaraldehyde, which creates stable bridges between pre-existing protein complexes, and oligomer formation was analysed by western blotting using Ab^cMyc^ (*Figure 1A*). Cross-linking of NT^Δβ8^^–^^β9^ resulted in the appearance of the tetramer; however, its abundance was extremely low relative to NT^WT^. Collective data (*n* ≥ 6) following densitometry analysis demonstrated that deletion of the β8–β9 loop resulted in substantial reduction (by 79% at 60 min) of the tetramer compared to WT (*Figure 1B*). NT^β13^^–^^β14/4Ala^, NT^Δβ20^^–^^β^21 and NT^Δβ31^^–^^β^32, the latter serving as negative control, produced oligomers to the same extent as NT^WT^, whereas deletion of the β23–β24 sequence resulted in reduced tetramer formation (by 39% at 60 min) that did not reach statistical significance compared to WT. These findings suggest a supporting role for the β23–β24 loop and identify the β8–β9 loop as the primary structural determinant for RyR2 N-terminus self-association.

**Figure 2:**
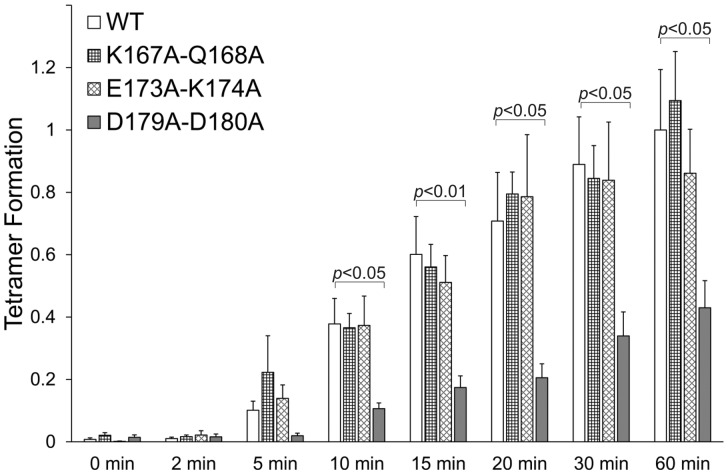
Aspartate 179 and aspartate 180 are indispensable for RyR2 N-terminus tetramerization. Chemical cross-linking assays (*n* = 8) of NT^WT^, NT^K167A + Q168A^, NT^E173A + K174A^, and NT^D179A + D180A^ as described in the legend to *Figure 1*. Data are given as mean value ± SEM; statistical analysis was carried out using Kruskal–Wallis test with Dunn’s multiple comparison test.

To pinpoint specific residues within the β8–β9 loop mediating N-terminus self-association, we consulted the recent high-resolution electron cryomicroscopy structures of RyR1 and RyR2.^5^^–^^10^. In spite of slight differences between the RyR1 and RyR2 structures and the lack of resolution for the side chain of some residues, RyR2 amino acids Q168, D179, and D180 appear to lie at the inter-subunit interface (Supplementary material online, *Table S3*). In particular, Q168 is in close proximity to the backbone of G239 (at the edge of the β13 strand) and the negatively charged side chain of D400 (within the β23–β24 loop). D179 and D180 are near to the positively charged side chain of H398, hydrophobic side chain of M399 and the peptide bond between these two residues (within the β23–β24 loop). We, therefore, generated separate NT constructs with double alanine substitutions of residues K167 + Q168, D179 + D180, and E173 + K174, the latter to serve as negative control for use in cross-linking assays (*n* = 8). As expected, NT^E173A^^+^^K174A^ produced tetramers equivalent to NT^WT^ demonstrating the specificity of our assay. Tetramer formation of NT^K167A^^+^^Q168A^ was similar to WT suggesting that putative interactions of the polar residue Q168 are of low affinity. On the other hand, the D179A + D180A mutant significantly decreased (by 57% at 60 min) tetramer formation (*Figure 2*). These results validate the data obtained with the β8–β9 loop deletion and demonstrate that the negatively-charged residues D179/D180 are necessary for efficient RyR2 N-terminus self-association.

### 3.2 The β8–β9 loop is required for RyR2 subunit oligomerization but dispensable for N-terminus interaction with C-terminus

**Figure 3:**
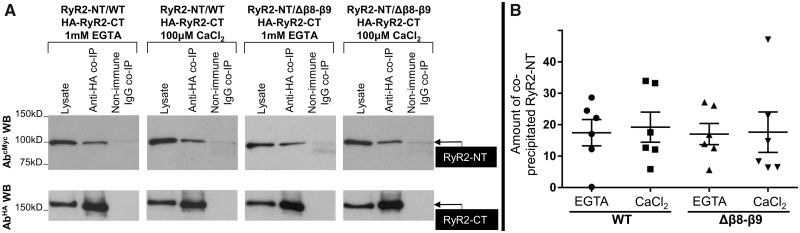
The β8–β9 loop is dispensable for RyR2 N-terminus interaction with C-terminus. Co-immunoprecipitation assays from HEK293 cells co-expressing NT^WT^ or NT^Δβ8–β9^ together with HA-tagged RyR2-CT (residues 3529–4967) in the presence of 1 mM EGTA or 100 μM CaCl_2_ as indicated. (*A*) HA-RyR2-CT was immunoprecipitated with Ab^HA^ from CHAPS-solubilized cell lysates and the presence of co-precipitated NT^WT^/NT^Δβ8–β9^ was analysed by western blotting using Ab^cMyc^ (top). To detect immuno-isolated HA-RyR2-CT, one-tenth of IP samples was analysed by western blotting using Ab^HA^ (bottom). Non-immune rabbit IgG served as negative control (non-specific binding). An aliquot of HEK293 cell lysate corresponding to 1% of the amount processed in the co-IP assay was included in the gels to assess protein expression. (*B*) Data summary (*n* = 6) for NT-specific binding (non-immune IgG IP signal subtracted from anti-HA IP signal) following densitometry analysis and normalization to each construct’s respective lysate (taken as 100%). Data are given as mean value ± SEM; statistical analysis was carried out using one-way analysis of variance with Bonferroni’s multiple comparisons test.

The near-atomic determination of the RyR1/2 structure revealed that sequences within the N-terminus, including the β8–β9 loop, may form several interfaces with sequences within the ‘core solenoid’ domain at the C-terminus.^5^^–^^10^ To reveal specific β8–β9 loop involvement in the putative N-terminus interaction with the pore-forming C-terminal region, we carried out co-IP experiments using HEK293 cells co-expressing NT^WT^ or NT^Δβ8^^–^^β9^ together with HA-tagged RyR2-CT (residues 3529–4967). Assays were carried out in nominally zero Ca^2+^ (1 mM EGTA) or its presence (100 μM CaCl_2_) due to potential Ca^2+^-binding site(s) within the C-terminus that may affect the interaction.^5^^,^^6^^,^^8^^,^^10^ HA-RyR2-CT was immunoprecipitated with Ab^HA^, verified by immunoblotting (*Figure 3A*, bottom panel), while the presence of co-precipitated NT^WT^ or NT^Δβ8^^–^^β9^ was analysed by western blotting using Ab^cMyc^. NT^WT^ was recovered in the HA immunoprecipitate but not in the negative control with non-immune rabbit IgG, irrespective of the presence (100 μM CaCl_2_) or absence (1 mM EGTA) of Ca^2+^ (*Figure 3A*). Similarly, NT^Δβ8^^–^^β9^ was efficiently co-immunoprecipitated with HA-RyR2-CT under both Ca^2+^-free and Ca^2+^-containing conditions. Quantitative data (*n* = 6) indicated RyR2-CT interaction with NT^Δβ8^^–^^β9^ are comparable to that with NT^WT^ and that this is unaffected by the presence of Ca^2+^ (*Figure 3B*). These results indicate a Ca^2+^-independent interaction between the RyR2 N- and C-termini that does not require the presence of the β8–β9 loop.

**Figure 4:**
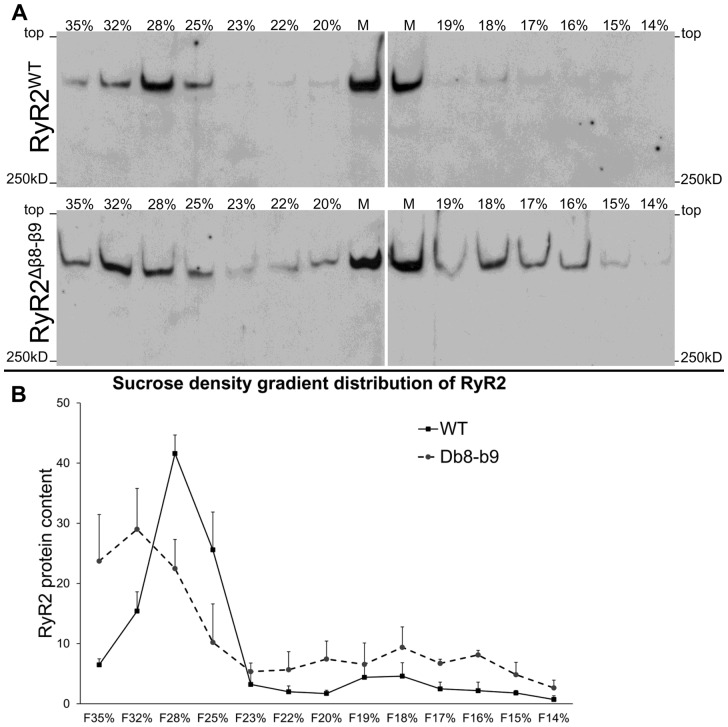
Deletion of the β8–β9 loop impairs RyR2 oligomerization. Protein distribution profile of RyR2^WT^ and RyR2^Δβ8–β9^ assessed by sucrose density gradient centrifugation. (*A*) RyR2^WT^ and RyR2^Δβ8–β9^ were expressed in HEK293 cells, CHAPS-solubilized microsomal membranes were subjected to density-gradient centrifugation and collected sucrose fractions were analysed by western blotting using RyR2 Ab^1093^. Microsomes (25 μg, M) were also included to assess protein expression; sucrose concentration as indicated. (*B*) Cumulative data (*n* = 4) following densitometry analysis and normalization against the amount of input protein in the microsomes; data are given as mean value ± SEM.

Given that N-terminus self-association is required for efficient oligomerization of full-length RyR2,^13^ we assessed the involvement of the β8–β9 sequence in RyR2 oligomerization by sucrose density gradient centrifugation. RyR2^WT^ and RyR2^Δβ8^^–^^β9^ (amino acids 167–178 removed) were expressed in HEK293 cells, CHAPS-solubilized microsomes were separated through linear 5–40% sucrose gradient, and gradient fractions were analysed by western blotting using Ab^1093^ (*Figure 4*). RyR2^WT^ was predominantly found in ‘heavy’ sucrose fractions (∼28%) consistent with tetramer formation. In contrast, RyR2^Δβ8^^–^^β9^ was found distributed throughout the gradient, a sedimentation profile indicative of weakly-associated tetramers in equilibrium with dissociated subunits.

### 3.3 The β8–β9 loop is required for RyR2 channel activation

**Figure 5:**
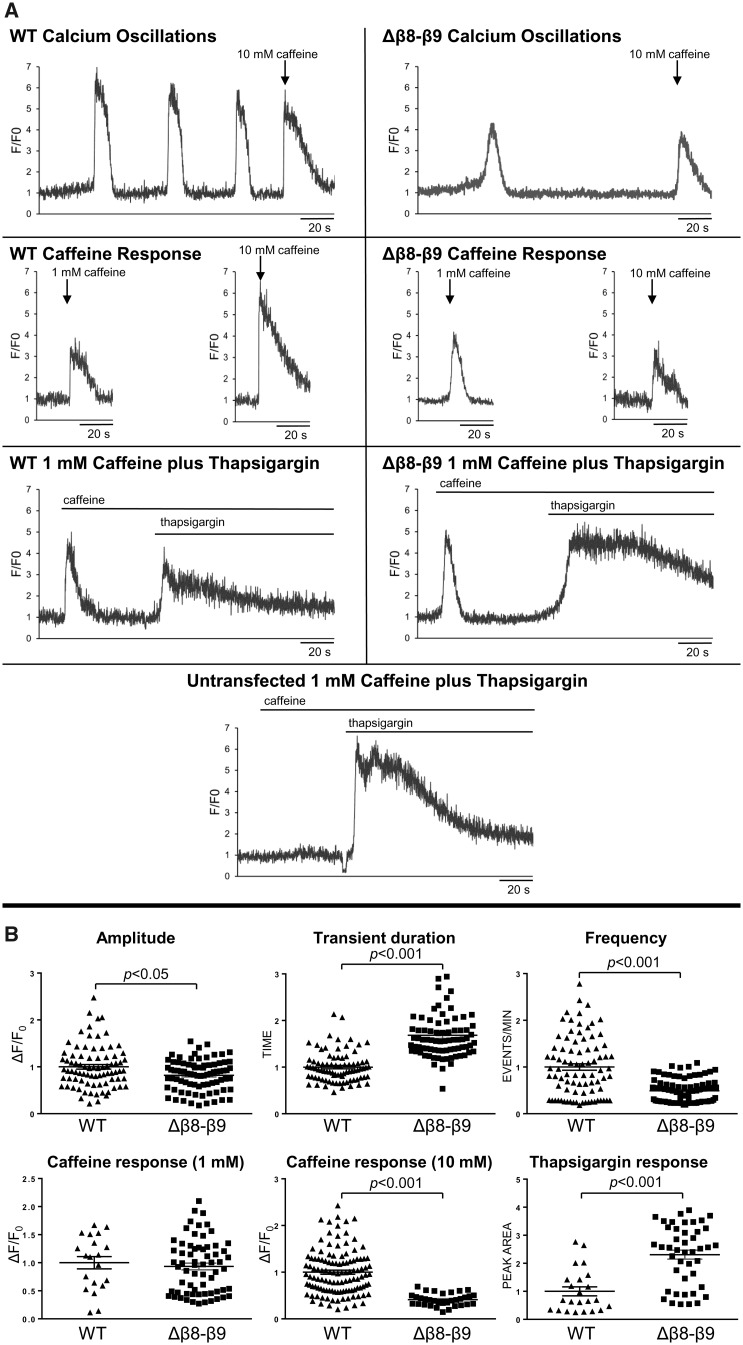
RyR2^Δβ8–β9^ displays drastically altered intracellular Ca^2^± release dynamics. Single-cell Ca2+ imaging using confocal laser scanning microscopy to monitor spontaneous Ca^2+^ release transient events. (*A*) Traces from Fluo-3 loaded single HEK293 cells expressing RyR2WT or RyR2^Δβ8–β9^ showing spontaneous Ca^2+^ transients and Ca^2+^ release induced by caffeine (1 mmol/L, 10 mmol/L) or thapsigargin (1 μmol/L). (*B*) Ca^2+^ transient characteristics, including amplitude, duration, and frequency of events, were analysed for 84 cells expressing RyR2^WT^ and 81 cells expressing RyR2^Δβ8–β9^ from four separate experiments, whereas the caffeine response for at least 20 cells. The ER Ca^2+^ store content was measured by integration of the (1 μmol/L) thapsigargin-induced Ca^2+^ release (following 1 mmol/L caffeine application) at the end of each experiment. Data are normalized for RyR2^WT^ and expressed as mean value ± SEM; statistical analysis was carried out using Mann–Whitney test.

RyR2^Δβ8^^–^^β^9 function was investigated within the intact cellular milieu using confocal microscopy and Ca^2+^ imaging to monitor spontaneous Ca^2+^ oscillations.^13^^,^^21^ Successful RyR2 expression was verified by immunofluorescence using Ab^1093^ indicating comparable transfection efficiency for RyR2^WT^ and RyR2^Δβ8^^–^^β^9 (Supplementary material online, *Figure S1*). Cells expressing RyR2^WT^ displayed the characteristic pattern of repetitive Ca^2+^ transients and also responded to suboptimal (1 mM) and maximal (10 mM) caffeine application (*Figure 5*). In contrast, cells expressing RyR2^Δβ8^^–^^β9^ exhibited very rare and diminished Ca^2+^ transients. Interestingly, RyR2^Δβ8^^–^^β9^-expressing cells had a similar response to WT when challenged with 1 mM caffeine but their response to maximal caffeine application was restrained compared to RyR2^WT^. The subdued RyR2^Δβ8^^–^^β^9 caffeine response did not allow for determination of the ER Ca^2+^ content, typically taken as the amplitude of the maximal caffeine-induced Ca^2+^ transient. To determine the relative ER Ca^2+^ content of cells expressing RyR2^WT^ and RyR2^Δβ8^^–^^β^9, we used the suboptimal caffeine dose to sensitize RyR2 (1 mM, a dose that produced comparable response between WT and mutant) followed by (1 μM) thapsigargin (inhibitor of the SR/ER Ca^2+^ ATPase pump) to empty the ER Ca^2+^ store, which was measured by integration of the resultant cytosolic Ca^2+^ transient. The summary of the Ca^2+^ imaging data presented in *Figure 5B* demonstrates that the RyR2 β8–β9 loop deletion drastically alters cellular Ca^2+^ cycling characteristics (Ca^2+^ transient amplitude, duration, frequency, and ER Ca^2+^ store). First, Ca^2+^ transient amplitude was reduced compared to WT, despite higher ER Ca^2+^ store, which could indicate that RyR2^Δβ8^^–^^β9^ is unable to open fully. Second, there was a significant decrease in the number of spontaneous Ca^2+^ oscillations compared to WT. This observation combined with the inability of caffeine to trigger maximum activation of the channel suggest that RyR2^Δβ8^^–^^β9^ exhibits profoundly altered sensitivity to agonists. Third, the ER Ca^2+^ store content was higher relative to WT, suggesting that RyR2^Δβ8^^–^^β9^ is much less prone to spontaneous channel opening than RyR2^WT^. Fourth, RyR2^Δβ8^^–^^β9^-expressing cells displayed a longer Ca^2+^ transient due to the larger Ca^2+^ store. These results indicate that residues 167–178 are crucial for RyR2 Ca^2+^ release.

As an alternative assay to assess the caffeine response of RyR2^Δβ8^^–^^β^9, we used high-affinity [^3^H]ryanodine binding of microsomal membranes. Experiments were conducted using HEK293 microsomes expressing equal amounts of RyR2^WT^ or RyR2^Δβ8^^–^^β^9 verified by immunoblotting (Supplementary material online, *Figure S2*) in the presence of 100 μM Ca^2+^ and 10 mM caffeine to promote maximum channel activation. Despite an equal amount of RyR2 protein, the β8–β9 loop deletion mutant displayed negligible [^3^H]ryanodine binding (2.0 ± 0.9 fmol/mg for RyR2^Δβ8^^–^^β9^ vs. 30.6 ± 2.0 fmol/mg for RyR2^WT^, *P *<* *0.01). Given that the caffeine and ryanodine-binding sites are located within the C-terminal transmembrane assembly,^5^ it is very unlikely that the small N-terminal deletion of residues 167–178 directly affects ligand binding. On the other hand, ryanodine (at nanomolar concentrations) binds only to intact RyR tetramers,^22^ which are compromised in RyR2^Δβ8^^–^^β^^9^ (*Figure 4*). Thus, the minute amount of bound [^3^H]ryanodine could only partly be due to diminished RyR2^Δβ8^^–^^β9^ response to agonists.

### 3.4 The arrhythmogenic R176Q mutation within the β8–β9 loop reduces N-terminus tetramerization and produces gain-of-function channels

**Figure 6:**
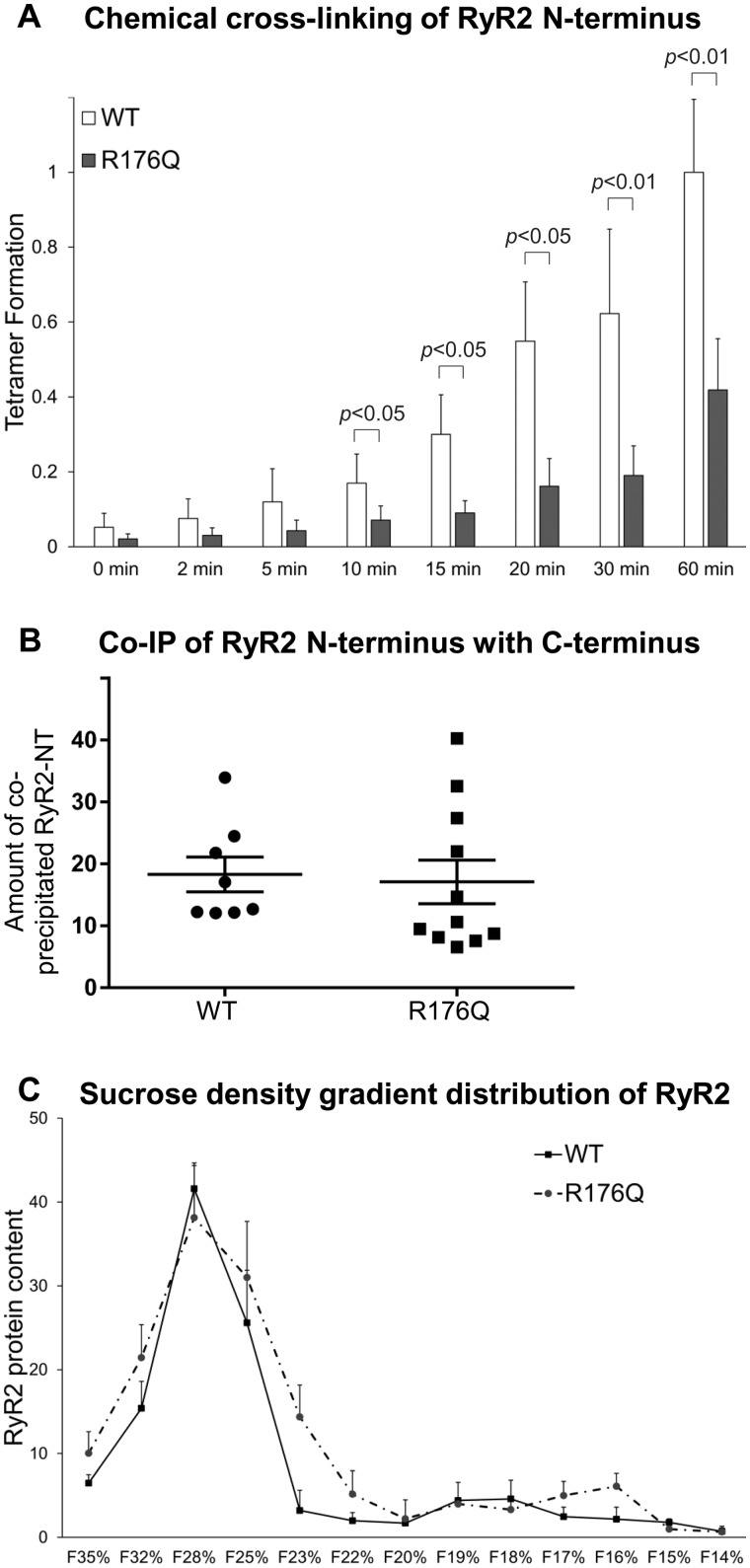
The pathogenic R176Q mutation decreases N-terminus tetramerization but it has no effect on N-terminus interaction with C-terminus or RyR2 tetramer assembly. (*A*) Chemical cross-linking assays (*n* = 8) of NT^WT^ and NT^R176Q^ as described in the legend to *Figure 1*. Data are given as mean value ± SEM; statistical analysis was carried out using Mann–Whitney test. (*B*) Co-immunoprecipitation assays (*n* ≥ 8) of HA-RyR2-CT with NT^WT^ or NT^R176Q^ as described in the legend to *Figure 3*. Data are given as mean value ± SEM; statistical analysis was carried out using Student’s t-test. (*C*) Sucrose density gradient centrifugation (*n* = 4) of RyR2^WT^ and RyR2^R176Q^ as described in the legend to *Figure 4*; data are given as mean value ± SEM.

To examine the involvement of the β8–β9 loop in RyR2 pathophysiology, we investigated the R176Q mutation linked with arrhythmogenic right ventricular dysplasia type 2 (ARVD2)^23^ and catecholaminergic polymorphic ventricular tachycardia (CPVT).^24^ Consistent with the results obtained with the β8–β9 loop deletion, chemical cross-linking analysis (*n* = 8) of NT^R176Q^ indicated reduced N-terminus tetramer formation (by 58% at 60 min) compared to WT (*Figure 6A*), and co-IP assays (*n* ≥ 8) demonstrated HA-RyR2-CT binding for NT^R176Q^ equivalent to NT^WT^ (*Figure 6B*). However, unlike RyR2^Δβ8^^–^^β^^9^ tetrameric channels which were unstable, sucrose density gradient centrifugation (*n* = 4) of RyR2^R176Q^ demonstrated full-length subunit oligomerization similar to WT (*Figure 6C*). These findings suggest that although the R176Q mutation disrupts N-terminus self-association, it has no effect on N-terminus interaction with C-terminus nor on RyR2 subunit oligomerization.

**Figure 7:**
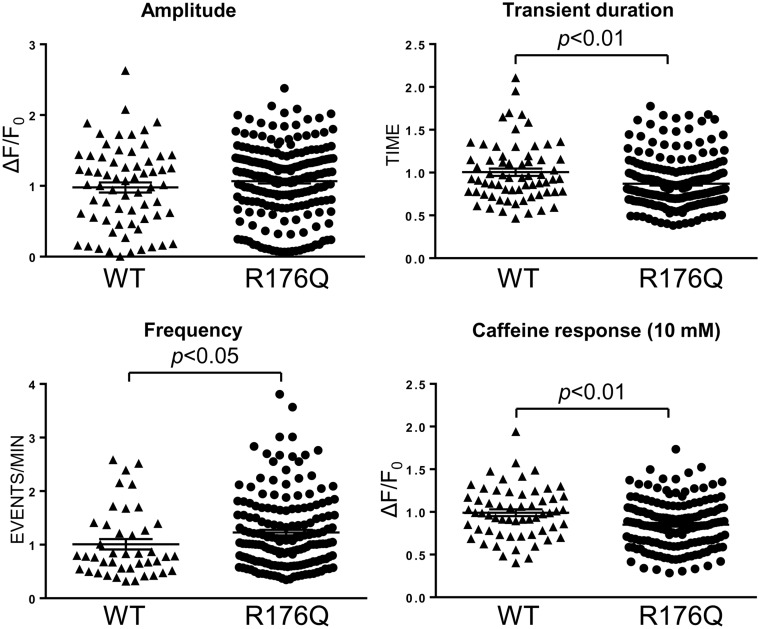
RyR2^R176Q^ displays hypersensitive channel characteristics. Single-cell Ca^2+^ imaging using confocal laser scanning microscopy to monitor spontaneous Ca^2+^ release transient events in HEK293 cells expressing RyR2^WT^ or RyR2^R176Q^ as described in the legend to *Figure 5*. Data from 66 cells expressing RyR2^WT^ and 222 cells expressing RyR2^R176Q^ from four separate experiments, are normalized for RyR2^WT^ and expressed as mean value ± SEM; statistical analysis was carried out using Mann–Whitney test.

The functional effects of the R176Q mutation were investigated by single-cell Ca^2+^ imaging to monitor spontaneous Ca^2+^ oscillations in intact cells. In contrast to RyR2^Δβ8^^–^^β^^9^ that produced restrained intracellular Ca^2+^ mobilization, RyR2^R176Q^-expressing cells displayed prominent Ca^2+^ oscillations (*Figure 7*). Compared to WT, RyR2^R176Q^ displayed a significant increase in the number of spontaneous Ca^2+^ transients with a concomitant smaller Ca^2+^ store, suggesting that the R176Q mutation results in hypersensitive and leaky channels. Ca^2+^ transient duration was smaller in RyR2^R176Q^-expressing cells most likely because there is less calcium available for release due to the smaller Ca^2+^ store.

## 4. Discussion

The N-terminus domain of RyR2, one of the three arrhythmia-associated mutation hot-spots, is an important structural and functional regulatory element involved in both activation and termination of SR Ca^2+^ release.^13^^,^^15^^,^^25^^–^^27^ The present study significantly extends previous findings by structurally defining the primary interaction sites mediating N-terminus self-association and also functionally demonstrates that the β8–β9 sequence is critically involved in RyR2 channel activity.

### 4.1 The β8–β9 loop is the primary determinant for RyR2 N-terminal inter-subunit interactions

High-resolution topological data^11^^,^^12^ enabled us to target discrete domains, namely the peptide sequences connecting the β8 strand with β9, β13 with β14, β20 with β21, and β23 with β24. We found that deletion of the β8–β9 loop severely compromises RyR2 N-terminus tetramerization, whereas deletion of the β23–β24 sequence results in a modest decrease (*Figure 1*). Conversely, deletion of the β20–β21 loop and the four-alanine substitution within the β13–β14 loop is largely without effect. These findings suggest that the β8–β9 loop is the principal oligomerization determinant, with the β23–β24 loop having a supporting role. Residues within the β13–β14 and β20–β21 loops do not appear to play a prominent role in N-terminus self-association; however, we cannot fully dismiss the contribution of these sequences due to limitations of our experimental approach. In particular, introduced deletions may not have been structurally disruptive due to compensatory structural rearrangements of neighbouring residues. This kind of protein plasticity has been previously reported in RyR2 for the disease-causative deletion of exon 3.^28^ Alternatively, deletion of an individual domain may not have been detrimental to N-terminus oligomerization due to the presence of additional determinants. The recent near-atomic electron cryomicroscopy maps of RyR1 and RyR2 confirm N-terminus tetrameric arrangement, which appears to be mediated through residues on a single inter-subunit interface that is qualitatively similar between these two RyR isoforms.^7^^,^^9^ The contact interface is likely formed by residues within the β8–β9 loop (and within the β2 sheet for RyR1 only) of subunit A, which are in close proximity (within 5 Å) with residues within the β13–β14 and β23–β24 loops (as well as within the β24 sheet for RyR1 only) of subunit B (Supplementary material online, *Table S3*). Using alanine substitution mutagenesis of the β8–β9 sequence, we found that the negatively charged RyR2 residues D179 and D180, which are closely opposed to amino acids H398 and M399 within the β23–β24 loop, perturbed N-terminus tetramerization (*Figure 2*). Notably, the D179N mutation has been linked with CPVT and the equivalent RyR1 residue (D166G and D166N) has been linked with malignant hyperthermia (Supplementary material online, *Figure S3*), further highlighting the pivotal involvement of the negatively charged side chain of this residue in N-terminus self-association. Thus, our empirical data together with the structural information demonstrate that the RyR2 N-terminal inter-subunit interaction is primarily mediated by amino acids 179–180 within the β8–β9 loop interacting with residues 398–399 within the β23–β24 loop of an adjacent subunit.

An additional finding of the present study is the demonstration of Ca^2+^-independent RyR2 N-terminus interaction with the C-terminal region encompassing the pore-forming transmembrane segments and the preceding structural element termed ‘core solenoid’.^5^^,^^7^^,^^9^ We have previously shown an interaction between the isolated N-terminus and the extreme C-terminal tail (residues 4867–4967) of RyR2^15^; however, the latter is buried within the context of the full-length protein,^5^^–^^10^ and therefore, this interaction does not confer physiological relevance for RyR2. Interestingly unlike RyR, the N-terminus and C-terminal tail of the related inositol trisphosphate receptor calcium release channel—which have high-peptide sequence and structural similarity with the corresponding RyR domains—are closely apposed in the context of the full-length channel, allowing for direct interaction to occur.^29^ In contrast, the high-resolution RyR1/2 structures indicate extensive contacts (within 5 Å) between the N-terminus and the core solenoid (residues 3620–4210).^5^^,^^7^^,^^9^ Interestingly, several disease-causative RyR1/2 mutations reside at these interfaces, suggesting a pathogenic role for altered association between N- and C-termini. Notably, the β8–β9 interface with the core solenoid is largely unaltered in the transition from the closed to the open state of the RyR2 channel, whereas the β8–β9 interface with the neighbouring N-terminus domain is widened in the open compared to the closed state^7^ (Supplementary material online, *Table S3*). The results presented here are consistent with the structural information and further indicate that the recently identified, C-terminal Ca^2+^-binding site^5^ is unlikely to affect N-terminus to C-terminus cross-talk. Importantly, the β8–β9 loop was found to be dispensable for N-terminus interaction with C-terminus (*Figure 3*) and, therefore, its role in RyR2 channel function seems to be primarily mediated through the regulation of N-terminal inter-subunit interactions.

The β8–β9 sequence is required not only for tetramerization of the N-terminus domain but also of the full-length RyR2 (*Figure 4*). This observation, which is consistent with our previous findings on the arrhythmogenic L433P mutation,^13^ highlights a crucial role for the N-terminal domain in RyR2 subunit oligomerization. Early work has demonstrated that the RyR C-terminal region containing the transmembrane domains forms a tetrameric cation-conducting pore that binds ryanodine.^30–32^ Moreover, the extreme C-terminal tail not only forms tetramers by itself^33^ but it is also essential for RyR subunit tetramerization.^34^ These studies together with the present results indicate that both the N-terminus and C-terminus domains are necessary—but insufficient on their own—for efficient RyR tetramer assembly.

### 4.2 The β8–β9 loop is indispensable for RyR2 function

Notably, the β8–β9 loop is a key functional element indicated by the dysfunctional RyR2^Δβ8^^–^^β^9 mutant. We found that deletion of residues 167–178 leads to defective channels that tend to remain inactive in living cells, indicated by subdued spontaneous Ca^2+^ oscillations in terms of both frequency and amplitude, and despite a higher ER Ca^2+^ content (*Figure 5*). These results are not surprising though, given that RyR2^Δβ8^^–^^β^9 displays reduced tetramer assembly with dissociated subunits unable to form Ca^2+^-conducting channels. The indispensable role of the β8–β9 sequence in channel function is highlighted by its extreme conservation across isoforms and species (including invertebrate RyR), as well as being the target of numerous RyR1/2 mutations associated with skeletomuscular and cardiac disease (Supplementary material online, *Figure S3*).

In this study, we biochemically and functionally characterized the arrhythmogenic R176Q mutation within the β8–β9 loop in RyR2. In agreement with previous reports,^35–37^ we found that RyR2^R176Q^ is a hypersensitive leaky channel displaying higher frequency of spontaneous Ca^2+^ transients and smaller intracellular Ca^2+^ store (*Figure 7*). To identify the molecular/structural defect(s), we carried out chemical cross-linking, co-immunoprecipitation, and gradient centrifugation analyses (*Figure 6*). We found that the R176Q mutation has the following effects: (i) weakens N-terminus tetramerization, (ii) has no effect on N-terminus interaction with C-terminus, and (iii) has no effect on full-length RyR2 subunit oligomerization. These findings indicate that disruption of N-terminal inter-subunit interactions is the primary mechanism underlying gain-of-function RyR2^R176Q^. Consistent with our results, previous solution nuclear magnetic resonance (NMR) and crystal structure studies showed that the R176Q mutation causes only local structural perturbations within the β8–β9 sequence proposed to affect the N-terminus inter-subunit interface.^38^

At first sight, our functional observations for RyR2^Δβ8^^–^^β^9 and RyR2^R176Q^, namely loss-of-function and gain-of-function channel, respectively, appear contradictory with each other. Both the β8–β9 deletion and R176Q mutation disrupt N-terminus tetramerization but the effect is more severe in the former, while neither manipulation alters N-terminus interaction with C-terminus. Notably though, unlike the R176Q mutation, the β8–β9 deletion also impairs full-length RyR2 subunit oligomerization, which accounts for the loss-of-function phenotype. Tellingly, the previously characterized L433P mutation, which also reduced both N-terminus tetramerization and full-length tetramer assembly, displayed both gain-of-function and loss-of-function characteristics.^13^ A closer look at the chemical cross-linking data (at 60 min) suggests a gradual effect in N-terminus tetramerization efficiency, which is in turn linked to tetrameric channel assembly, with the two biochemical defects having contrasting functional effects. RyR2^WT^ has robust N-terminus tetramerization stabilizing the closed conformation of intact tetrameric channels. Moderate disruption (by 58%) of N-terminus tetramerization but retaining intact full-length tetramers in RyR2^R176Q^, is manifested as a gain-of-function channel. Severe disruption (by 79%) of N-terminus tetramerization resulting in impairment of full-length tetramer assembly in RyR2^Δβ8^^–^^β^9, is manifested as loss-of-function channel. RyR2^L433P^ with reduced (by 67%) N-terminus tetramerization and concomitant impairment of full-length tetramer assembly lies in between the two, displaying characteristics of both hypersensitive and hyposensitive channel.^13^

Our present findings implying that the β8–β9 domain is involved in both opening and closing of the channel, are compatible with previous reports demonstrating that the N-terminus governs both activation and termination of SR Ca^2+^ release.^13^^,^^15^^,^^25–27^ Moreover, they have important clinical implications. While the vast majority of RyR2 mutations produce hypersensitive channels with increased diastolic SR Ca^2+^ leak resulting in delayed after-depolarizations, there is a handful of distinct RyR2 mutations including the ARVD2-linked L433P^13,^^37^ and the hypertrophic cardiomyopathy-linked A1107M mutation reported to reduce Ca^2+^ release.^27^ In addition, the CPVT-linked A4860G mutation was found to depress RyR2 channel activity,^39^ whereas ventricular myocytes from A4860G knockin mice had reduced amplitude of systolic Ca^2+^ release with overloaded SR Ca^2+^ content, which randomly caused bursts of prolonged systolic Ca^2+^ release triggering early after-depolarizations.^40^ Thus, it is conceivable that drugs that target gain-of-function RyR2 mutations, while beneficial for the majority of patients, may have deleterious effects on patients harbouring loss-of-function mutations.

### 4.3 Limitations

The present study made use of targeted removal of peptide loops connecting neighbouring β strands and despite being designed following careful bioinformatics analysis of the available structural data, we cannot exclude the possibility of local or global conformational changes. The functional observations seen with deletion of residues 167–178 could be due to altered global conformation of the channel—although the remaining three residues within this loop would be expected to bridge the β8 and β9 strands. Moreover, the deletion did not affect the N-terminus interaction with the C-terminus arguing against global conformational destabilization. Thus, it is likely that RyR2^Δβ8^^–^^β9^ dysfunction is due to the specific loss of one of the contact sites at the N-terminus inter-subunit interface, which in turn perturbs the assembly of functional tetrameric channels.

The present work employed RyR2-expressing HEK293 cells because the study of genetically manipulated RyR2 is not feasible in the native context unless animal knockin models are available. At present, there is no efficient methodology to deliver the very large (∼15 kb) RyR2 cDNA inside cardiac myocytes, whereas gene editing is generally believed to be feasible only for actively dividing cells. A recent study has indicated that homology-directed repair is feasible in non-dividing cardiac myocytes in culture; however, gene editing was of low efficiency (up to 20–25%) for cardiac myocytes at neonatal stage, and it was not observed in adult cells.^41^ The RyR2-expressing HEK293 system is an experimental cellular assay that recapitulates spontaneous Ca^2+^ release under Ca^2+^ overload conditions in cardiac myocytes.^20^^,^^42^ Enhancement of RyR2 activity with caffeine^43^^,^^44^ or CPVT gain-of-function mutations (e.g. R4496C)^20^^,^^45^^,^^46^ result in higher frequency of spontaneous Ca^2+^ transients and lower Ca^2+^ store content in both HEK293/RyR2 cells and cardiac myocytes. One discrepancy is that Ca^2+^ transient amplitude is largely unaffected in HEK293/RyR2 cells, whereas it is decreased in cardiac myocytes implicating the involvement of cardiac-specific accessory proteins.

## 5. Summary

We identify a short peptide sequence, which, although located remotely from the pore in terms of both primary and tertiary structure, is vital for RyR2 channel gating. The empirical findings presented in this study together with the known structural information allow us to draw a plausible hierarchical order of events leading to RyR2 channel activation. Efficient inter-subunit N-terminus to N-terminus communication mediated by the β8–β9 loop is likely to constitute a primary signal for RyR2 gating. This may then be transmitted in a Ca^2+^-insensitive manner to the core solenoid domain via N-terminal sequences other than the β8–β9 residues, and subsequently conveyed on to the C-terminal channel domain to induce gating of the pore. Perturbation of N-terminal inter-subunit interactions, solely or in combination with impairment of RyR2 tetramer assembly, can lead to either gain-of-function or loss-of-function channels, respectively.

## Supplementary material

Supplementary material is available at *Cardiovascular Research* online.

**Conflict of interest:** none declared.

## Funding

This work was supported by a British Heart Foundation Fellowship [FS/15/30/31494 to S.Z.] and a British Heart Foundation project grant [PG/16/92/32453 to N.L.T.].

## Supplementary Material

cvaa043_Supplementary_Data

## Notes

**Time for primary review: 20 days**

## Notes

Translational perspective Our findings that the ryanodine receptor 2 (RyR2) β8–β9 loop is involved in both Ca^2+^ release channel opening and closing have important clinical implications. This RyR2 domain is a known ‘hot-spot’ for mutations associated with arrhythmogenic cardiac disease, which could produce hypersensitive and hyposensitive channels. Therapeutic strategies currently focus on gain-of-function RyR2 channels to suppress sarcoplasmic reticulum Ca^2+^ release either indirectly with Class I/II anti-arrhythmic drugs, or by directly targeting RyR2 to inhibit channel activity. These strategies may not only be ineffective but also they may exacerbate the malignant phenotype in the case of loss-of-function RyR2 mutations.
