# Supplementary material for: Identification of an amino-terminus determinant critical for ryanodine receptor/Ca2+ release channel function
Source: Cardiovasc Res. 2020 Feb 20;117(3):780–91. doi: 10.1093/cvr/cvaa043 (PMC7898959; doi:10.1093/cvr/cvaa043)
Supplement: cvaa043_Supplementary_Data [file cvaa043_supplementary_data.docx]

**Identification of an amino-terminus determinant critical for ryanodine receptor/Ca^2+^ release channel function**

Short title: A ryanodine receptor domain vital for Ca^2+^ release

Monika Seidel^a,b^, Camille Rabesahala de Meritens^a,b^, Louisa Johnson^a,b^, Dimitris Parthimos^a,c^, Mark Bannister^a,b^, N. Lowri Thomas^a,d^, Esizaze Ozekhome-Mike^a,b^, F. Anthony Lai^e^, Spyros Zissimopoulos^a,b^

^a^Wales Heart Research Institute, Cardiff University, Cardiff, CF14 4XN, UK

^b^Swansea University Medical School, Institute of Life Science, Swansea, SA2 8PP, UK

^c^School of Medicine, Division of Cancer & Genetics, Cardiff University, Cardiff, CF14 4XN, UK

^d^School of Pharmacy & Pharmaceutical Sciences, Cardiff University, Cardiff, CF10 3NB, UK

^e^College of Medicine, QU Health, Qatar University, Doha, Qatar

**SUPPLEMENTARY MATERIAL**

**Supplementary Figures**


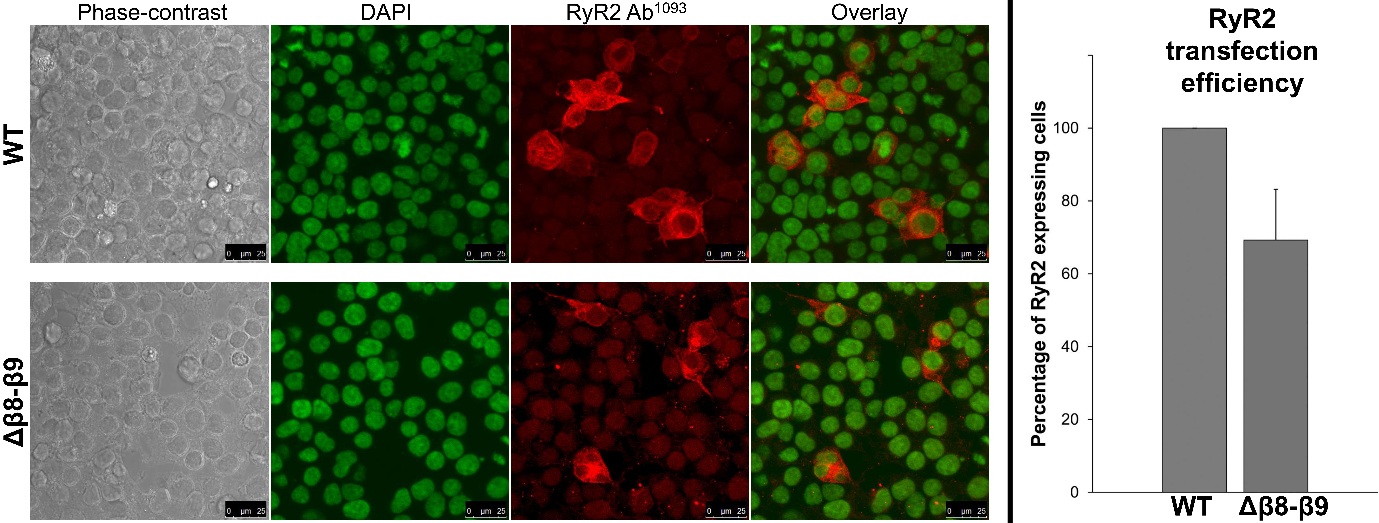


Supplemental Figure 1: Comparable transfection efficiency for RyR2^WT^ and RyR2^Δβ8-β9^

**Left.** Immunofluorescence of HEK293 cells transfected with RyR2^WT^ or RyR2^Δβ8-β9^ using Effectene (Qiagen). 48 hr post transfection, cell nuclei were stained with (1ng/μl) DAPI and RyR2 was immunolocalised with (1:100 dilution) Ab^1093^ and Alexa^594^-conjugated anti-rabbit antibody (1:500 dilution) for fluorescent labelling.

**Right.** Cumulative data (n = 4) for RyR2-positive cells presented as percentage of DAPI-stained cells and normalized for WT. Data are given as mean value ± SEM; statistical analysis was carried out using Student’s t-test (*p* = 0.118).





Supplemental Figure 2: Equivalent protein expression for RyR2^WT^ and RyR2^Δβ8-β9^

**A.** Western blot analysis of HEK293 microsomal membranes (100μg) expressing RyR2^WT^ or RyR2^Δβ8-β9^ using Ab^1093^.

**B.** Cumulative data following densitometry analysis from three separate experiments; data are given as mean value ± SEM.

β9

β8-β9 loop

β8

RyR1 Pig MTDKLAFDVG LQEDATGEAC WWTTHPASKQ RSEGEKVRVG DDLILVSVSS

RyR1 Human MTDKLAFDVG LQEDATGEAC WWTMHPASKQ RSEGEKVRVG DDIILVSVSS

RyR1 Rabbit MTDKLAFDVG LQEDATGEAC WWTMHPASKQ RSEGEKVRVG DDLILVSVSS

RyR1 Fish LTDKLAFDVG LQEDSTGEAC WWTIHPASKQ RSEGEKVRVG DDLILVSVSS

RyR1 Frog LTDKLAFDVG LQEDASGEAC WWTIHPASKQ RSEGEKVRIG DDLILVSVSS

RyR2 Rabbit STDKLAFDVG LQEDTTGEAC WWTIHPASKQ RSEGEKVRVG DDLILVSVSS

RyR2 Human STDKLAFDVG LQEDTTGEAC WWTIHPASKQ RSEGEKVRVG DDLILVSVSS

RyR2 Mouse STDKLAFDVG LQEDTTGEAC WWTIHPASKQ RSEGEKVRVG DDLILVSVSS

RyR3 Human QTDKLAFDVG LREHATGEAC WWTIHPASKQ RSEGEKVRIG DDLILVSVSS

RyR3 Rabbit QTDKLAFDVG LREHATGEAC WWTIHPASKQ RSEGEKVRIG DDLILVSVSS

RyR3 Mink QTDKLAFDVG LRENATGEAC WWTIHPASKQ RSEGEKVRIG DDLILVSVSS

RyR3 Chicken QTDKLAFDVG LRENAAGEAC WWTIHPASKQ RSEGEKVRIG DDLILVSVSS

RyR3 Frog LTDKLAFDVG LQDTATGEAC WWTIHPASKQ RSEGEKVRIG DDLILVSVSS

RyR SeaUrchin SKDKLAFDVG LQESTQGEAC WWTIHPVSKQ RSEGEKVRVG DDLILVNVAT

RyR Fly SNDKLSFDVG LQEHSQGEAC WWTVHPASKQ RSEGEKVRVG DDLILVSVAT

RyR Nematode SNDKLAFDVG VQETNEGEAC WWTIHPASKQ RSEGEKVRVG DDVILVSVAT

Supplementary Figure 3: Conservation of RyR β8-β9 sequence

RyR1 mutations associated with Central Core Disease and Malignant Hyperthermia:

1. Q155K (Robinson *et al*., Hum Mutat, 2006) & (Ibarra *et al*., Anesthesiology, 2006)
2. R156K (Galli *et al*., Hum Mutat, 2006)
3. E160G (Shepherd *et al*., J Med Genet, 2004), (Robinson *et al*., Hum Mutat, 2006) & (Miller *et al*., Br J Anaesth, 2018)
4. R163C (Quane *et al*., Nat Genet, 1993), (Monnier *et al*., Anesthesiology, 2002) (Rueffert *et al*., Acta Anaesthesiol Scand, 2002), (Ibarra *et al*., Anesthesiology, 2006) & (Brandom *et al*., Anesth Analg, 2013)
5. R163L (Monnier *et al*., Hum Mutat, 2005) & (Robinson *et al*., Hum Mutat, 2006)
6. G165R (Monnier *et al*., Hum Mutat, 2005)
7. D166N (Rueffert *et al*., Acta Anaesthesiol Scand, 2002) & (Monnier *et al*., Hum Mutat, 2005)
8. D166G (Robinson *et al*., Hum Mutat, 2006) & (Ibarra *et al*., Anesthesiology, 2006)

RyR2 mutations associated with Arrhythmogenic Right Ventricular Dysplasia type 2, Catecholaminergic Polymorphic Ventricular Tachycardia type 1, Dilated Cardiomyopathy and Long QT Syndrome:

1. P164S (Choi *et al*., Circulation, 2004), (Tester *et al*., Heart Rhythm, 2006), (Medeiros-Domingo *et al*., J Am Coll Cardiol, 2009) & (Lin *et al*., J Electrocardiol, 2018)
2. A165D (Xiong *et al*., J Mol Cell Cardiol, 2018)
3. S166C (Haas *et al*., Eur Heart J, 2014) & (Shigemizu *et al*., PLoS One, 2015)
4. R169L (Ohno *et al*., PLoS One, 2015) & (Miyata *et al*., Intern Med, 2018)
5. R169Q (Hsueh *et al*., Int J Cardiol, 2006), (Kawamura *et al*., Circ J, 2013), (Ohno *et al*., PLoS One, 2015) & (Miyata *et al*., Intern Med, 2018)
6. R176Q (Tiso *et al*., Hum Mol Genet, 2001), (Bauce *et al*., J Am Coll Cardiol, 2002), (Tester *et al*., Heart Rhythm, 2005) & (Haugaa *et al*., Europace, 2010)
7. G178A (Ohno *et al*., PLoS One, 2015) & (Miyata *et al*., Intern Med, 2018)
8. D179N (Kawata *et al*., Circ J, 2016)

**Supplementary Tables**

Supplementary Table 1: PCR primer information for RyR2 NT deletion constructs

Coordinates based on the human RyR2 cDNA sequence (GenBank accession number: X98330); * phosphorothioate modified nucleotides, ¶ β13-β14/4Ala is a substitution for four alanine residues.

| Construct | deletionCoordinates | Primers |
| --- | --- | --- |
|  |  |  |
| Δβ8-β9 | 167-178 | gcctctgatgac *c*t*c*a tcttagtt  GTCATCAGAGGC *A*G*G*G TGTATGGT |
| β13-β14  /4Ala ^¶^ | 240-243 | CCTCAGGTTGCTGCATGGAGCCGCGGCCGCGTGTCTCACTGTCCCTTCAGG  CCTGAAGGGACAGTGAGACACGCGGCCGCGGCTCCATGCAGCAACCTGAGG |
| Δβ20-β21 | 335-358 | Tcttcctcagta *t*g*c*t atatacaacatgtagacacaggcctatgg  tactgaggaaga *c*c*g*g aaggtaaatgctgttgattttacatcaga |
| Δβ23-β24 | 399-401 | ATGCATCATGAAGGCCACGGCATAAGTTTGTCGAGA  TCTCGACAAACTTATGCCGTGGCCTTCATGATGCAT |
| Δβ31-β32 | 748-752 | caacatgatgtc *a*t*c*a gttgctgtttagatctgagtgccccaagc  gacatcatgttg *g*t*t*t ggtgagcttacagtacgagcaatacaacc |

Supplementary Table 2: RyR2 NT deletion constructs used in chemical cross-linking assays

Coordinates based on the human RyR2 peptide sequence (GenBank accession number: X98330); * β13-β14/4Ala: indicated peptide sequence has been substituted for alanine residues.

| **NT construct** | **Targeted structural element** | **Deleted peptide sequence** | **Deletion coordinates** |
| --- | --- | --- | --- |
|  | | | |
| **Δβ8-β9** | β8-β9 loop (165-179) | KQRSEGEKVRVG | 167-178 |
| **β13-β14/4Ala ^*^** | β13-β14 loop (240-243) | HMDE | 240-243 |
| **Δβ20-β21** | β20-β21 loop (333-359) | KEKLDVGVRKEVDGMGTSEIKYGD | 335-358 |
| **Δβ23-β24** | β23-β24 loop (395-402) | MDD | 399-401 |
| **Δβ31-β32** | β31-β32 loop (740-753) | LLRTD | 748-752 |

Supplementary Table 3: Atomic distance in the open versus the closed RyR state

| RyR2 N-Terminus to N-TerminusInter-Subunit Interface | | |
| --- | --- | --- |
|  | | |
| **Residues** | **Closed State**  **Distance** | **Open State**  **Distance** |
| S166 – D400 | 8.5Å | 11.8Å |
| Q168 – G239 | 5.4Å | 7.2Å |
| Q168 – D400 | 5.2Å | 8.9Å |
| D179 – M399 | 10.1Å | 13.2Å |
| D180 – H398 | 9.0Å | 12.4Å |
| D180 – M399 | 7.7Å | 11.0Å |

| RyR2 β8-β9 loop to C-TerminusInter-Subunit Interface | | |
| --- | --- | --- |
|  | | |
| **Residues** | **Closed State**  **Distance** | **Open State**  **Distance** |
| S170 – R3939 | 6.8Å | 8.8Å |
| E171 – L3879 | 5.4Å | 4.8Å |
| E171 – E3883 | 4.0Å | 4.3Å |
| E171 – D3887 | 5.2Å | 8.2Å |
| G172 – E3883 | 3.7Å | 3.4Å |
| E173 – R3939 | 4.9Å | 5.1Å |
| K174 – D3942 | 4.4Å | 3.9Å |
| K174 – E4049 | 3.2Å | 5.7Å |

Atomic distance between listed residues taken from the cryo-electron microscopy structures of RyR1 in the closed (5TB0) and open state (5TAL), and of RyR2 structures in the closed (5GO9) and open state (5GOA). RyR C-terminus residues in close proximity to β8-β9 loop only are given. For comparison, we have included the distance between RyR2 residues in the N-terminus to N-terminus interface, which is within 5Å between the corresponding RyR1 residues.

| RyR1 N-Terminus to N-TerminusInter-Subunit Interface | | |
| --- | --- | --- |
|  | | |
| **Residues** | **Closed State**  **Distance** | **Open State**  **Distance** |
| S153 – D384 | 4.6Å | 10.8Å |
| Q155 – G224 | 4.8Å | 7.4Å |
| Q155 – H225 | 5.1Å | 4.9Å |
| Q155 – D384 | 4.7Å | 8.6Å |
| Q155 – A386 | 4.5Å | 9.3Å |
| G165 – M383 | 3.3Å | 7.6Å |
| D166 – M383 | 3.6Å | 8.9Å |
| D167 – H382 | 5.8Å | 12.9Å |
| D167 – M383 | 4.0Å | 8.2Å |

| RyR1 β8-β9 loop to C-TerminusInter-Subunit Interface | | |
| --- | --- | --- |
|  | | |
| **Residues** | **Closed State**  **Distance** | **Open State**  **Distance** |
| S157 – R3983 | 4.2Å | 6.3Å |
| E158 – E3927 | 4.4Å | 5.8Å |
| E158 – R3983 | 4.2Å | 5.0Å |
| G159 – E3927 | 5.2Å | 4.8Å |
| G159 – R3983 | 6.2Å | 3.5Å |
| G159 – D3986 | 2.9Å | 5.0Å |
| E160 – R3983 | 2.6Å | 4.0Å |
| K161 – D3986 | 4.0Å | 4.0Å |
| K161 – E4049 | 4.0Å | 7.1Å |
